# The Inhibitory T Cell Receptors PD1 and 2B4 Are Differentially Regulated on CD4 and CD8 T Cells in a Mouse Model of Non-alcoholic Steatohepatitis

**DOI:** 10.3389/fphar.2019.00244

**Published:** 2019-03-13

**Authors:** Cordula Hansel, Stephanie Erschfeld, Maike Baues, Twan Lammers, Ralf Weiskirchen, Christian Trautwein, Daniela C. Kroy, Hannah K. Drescher

**Affiliations:** ^1^Department of Internal Medicine III, University Hospital Aachen, RWTH Aachen University, Aachen, Germany; ^2^Institute for Experimental Molecular Imaging, University Hospital Aachen, RWTH Aachen University, Aachen, Germany; ^3^Institute of Molecular Pathobiochemistry, Experimental Gene Therapy, and Clinical Chemistry (IFMPEGKC), University Hospital Aachen, RWTH Aachen University, Aachen, Germany

**Keywords:** Western diet, inhibitory T cell receptors, non-alcoholic steatohepatitis, NASH, liver, programmed cell death protein 1, natural killer cell receptor 2B4

## Abstract

Infiltrating CD4 and CD8 T cells have been shown to worsen inflammatory liver damage in non-alcoholic steatohepatitis (NASH). Inhibitory T cell receptors such as the programmed cell death protein 1 (PD1) and the natural killer cell receptor 2B4 regulate the activity of CD4 and CD8 T cells and therefore play an important role in immune tolerance required in the liver. In this study, we investigated the expression profile of inhibitory T cell receptors on CD4 and CD8 T cells in a mouse model of NASH. Male B57BL/6J mice were fed a Western diet for 24 weeks. The expression levels of inhibitory receptors on the surface of intrahepatic and peripheral T cells were measured and correlated with markers of activation (CD107a, CD69, and CD44), metabolic disorder (serum triglycerides, serum cholesterol, γ-glutamyl transferase, hepatic triglycerides), inflammation (serum alanine aminotransferase and aspartate aminotransferase) and hepatic fibrosis (collagen 1A1, α-smooth muscle actin, hydroxyproline). Under Western diet, PD1 is exclusively upregulated on intrahepatic and peripheral CD8^+^ T cells, whereas the expression level on CD4 T cells is unaffected. In contrast, 2B4 is upregulated liver-specifically on both CD4 and CD8 T cells and unchanged on peripheral T cells. Upregulation of PD1 on CD8 T cells is restricted to CD8 effector memory T cells and correlates with lower levels of degranulation. Similarly, the inhibitory function of PD1 on intrahepatic CD4 T cells is shown by a lower CD69 and CD44 expression on PD1-positive CD4 T cells. In murine steatohepatitis, the upregulation of PD1 on CD8 T cells and 2B4 on CD4 and CD8 T cells potentially limits T cell-mediated liver damage. Therefore, these inhibitory T cell receptors could serve as promising targets of immune-modulatory NASH therapy.

## Introduction

Non-alcoholic fatty liver disease is the most common hepatic disease in developed countries and is mostly accompanied by further manifestations of the metabolic syndrome like type 2 diabetes, hypertension, obesity and dyslipidemia. In an American study performed by proton magnetic resonance spectroscopy of the liver, the frequency of fatty liver was calculated as 30%, depending on the ethnical background with the highest frequency in Hispanics (45%) and the lowest frequency in African American (24%) (Mishra and Younossi, 2012). NAFLD with isolated steatosis may easily progress to more advanced stages like NASH in 30%, which can end up in cirrhosis in up to 20% (Mishra and Younossi, 2012). As a consequence of cirrhosis complications like variceal bleeding, hepatocellular carcinoma, spontaneous bacterial peritonitis or hepato-renal syndrome as well as the concomitantly augmented risk for cardiovascular events, NASH patients have a markedly reduced life expectancy (Ong and Younossi, 2007). According to another large United States study, examining data from 37500 adults, the prevalence of NAFLD duplicated within 20 years (Younossi et al., 2011; Younossi et al., 2018a,b). Consequently, NASH-related decompensated cirrhosis is predicted to increase by 168% to more than 100.000 cases in the United States and thereby evolve to the leading cause for liver transplantation until 2030 (Afzali et al., 2012; Estes et al., 2018). In spite of this increasing socio-economical challenge, no effective pharmacological therapy is currently available. Therefore, a better understanding of the mechanisms promoting the progression from simple steatosis to steatohepatitis is crucial.

In 1998 Day and James proposed the “two hit hypothesis” of NASH pathogenesis, postulating a first hit of lipid accumulation in hepatocytes entailing oxidative stress and apoptosis. This steatosis sensitizes the liver to various second hits created by infiltrating immune cells and their harmful soluble mediators creating an inflammatory environment and finally stimulating fibrotic remodeling (Day and James, 1998). Hence, infiltrating immune cells are an important pre-condition for developing advanced NASH. So far, in human NASH, most studies focused on the role of the *innate immune system* in NASH. However, little is known about the influence and the properties of infiltrating T cells in human steatohepatitis. Following Ma et al., liver specimen from NASH and ASH patients showed a moderate CD8 T cell infiltrate, but fewer CD4 T cells and a lower CD4/CD8 ratio than serum ALT- and AST-matched specimen from viral hepatitis specimen (Bohne et al., 2014; Ma et al., 2016). In other human liver diseases characterized by liver steatosis, such as ASH and chronic HCV genotype 3 infection, hepatic inflammation is accompanied by an increased CD8 T cell infiltrate as well (Wolf et al., 2014). Recently, several mouse models of NASH confirmed an important role of intrahepatic T cells for NASH progression. In mice fed a methionine- and choline-deficient diet (MCD) the starting intrahepatic T cell number was 10% of total intrahepatic leukocytes and their absolute cell number was tripled under MCD (Henning et al., 2013). In a CD-HFD mouse model, CD8 T cells showed an activated phenotype and mice that genetically lacked T cells (Rag1^-/-^, β2m^-/-^) were protected from NASH (Wolf et al., 2014). Furthermore, two studies described a harmful role for CD8 T cells in adipose tissue inflammation, which subsequently deteriorated histological findings in NASH (Nishimura et al., 2009; Popov and Schuppan, 2010). In contrast, regulatory T cells seem to play a protective role by suppressing CD4 and CD8 T cells in steatotic liver, as their depletion in HFD fed mice was associated with increased inflammation. Their number was reduced in fatty liver because of increased susceptibility to oxidative stress-induced apoptosis compared to other T cell subclasses (Ma et al., 2007). Given these results we assume a harmful role for CD4^+^ and especially CD8^+^ T cells in NASH pathogenesis and a potential impact of regulatory T cell receptors on NASH severity.

Inhibitory and activating T-cell receptors fine-tune T-cell responses to fight microorganisms and carcinoma cells while avoiding autoimmunity. Inadequate stimulatory and inhibitory signals can result either in a non-sufficient activation level of T cells, that fail to eliminate microbiological pathogens and degenerated cells or an over-activation of T cells, leading to immune mediated self-damage.

Initially, inhibitory T cell receptor ligands, especially the PD-L1, have been found to be expressed by different tumor cell lines to evade the surveillance of host T cells (Ohigashi et al., 2005; Nakanishi et al., 2007; Droeser et al., 2013). In this context, inhibitory PD1 antibodies like Nivolumab^®^ and Pembrolizumab^®^ have been successfully introduced as immunotherapy of non-small-cell lung cancer, melanoma and urothelium cancer (Herbst et al., 2016; Johnson et al., 2016; Rosenberg et al., 2016). In the context of chronic liver diseases, the inhibitory T cell receptors PD1 and 2B4 were intensively investigated in chronic HBV and HCV infection, where their upregulation could support viral persistence (Bohne et al., 2014; Owusu et al., 2015; Tang et al., 2016). Moreover, PD1 plays an important role in non-infectious liver diseases like biliary obstruction in mice (Licata et al., 2013), autoimmune hepatitis (Matsumoto et al., 2014) and acute alcoholic hepatitis (Markwick et al., 2015). However, their impact on metabolic diseases has been unattended to date. Some studies dealt with the influence of PD1 on autoimmune diabetes type 1, describing a disease modification after administration of anti-PD1 antibody (Kochupurakkal et al., 2014; El Khatib et al., 2015; Gaudy et al., 2015; Lee et al., 2015) or a correlation with genetic polymorphisms of PD1 (Lee et al., 2015).

Therefore, our study addresses the question, whether the inhibitory T cell receptors PD1 and 2B4 are upregulated and potentially limit T cell mediated liver damage in NASH. Prospectively, these receptors could serve as new pharmacological targets of antibody-based therapy of steatohepatitis.

## Materials and Methods

### Mice

All experiments were admitted by the local authority, which is the Landesamt für Natur, Umwelt und Verbraucherschutz Nordrhein-Westfalen (LANUV), and followed the criteria of the German administrative panel of laboratory animal care. Male wild-type BL/6J mice at the age of 8–9 weeks were fed a WD for 24 weeks. As controls, mice were fed a chow diet for the same period of time. The WD was produced by Brogaarden, D09100301 and consists of 40% kcal fat, 20% kcal fructose and 2% kcal cholesterol (Supplementary Figure 1). The exact composition of both diets is listed in Supplementary Figure 7. Mice experienced 12-h light/dark cycles, had free access to food and drinking water. In all experiments at least 5 animals per group were analyzed.

### Glucose Tolerance Test (GTT)

After fasting for 6 h sober blood glucose was measured. After administration of 2 g/kg glucose intraperitoneally blood glucose levels were measured every 15 min for 2 h.

### Serum Analysis

Serum aspartate aminotransferase (ST), ALT cholesterol and triglyceride levels were measured in the University Hospital RWTH Aachen Central Laboratory Facility.

### Hepatic Triglycerides

Hepatic triglycerides were measured in 20 mg liver tissue. Tissue was homogenized in 1 ml homogenization buffer (10 mM Tris, 2 mM EDTA, 0.25 M sucrose, pH 7.5). The standard curve was prepared using the Instruchemie liquicolor Kit (Instruchemie, Delfzijl, Netherlands). 200 μl kit reagent were added to 2 μl tissue homogenate. After 45 min incubation at room temperature OD was measured at 492 nm.

### Intrahepatic Gene Expression Analysis via Real-Time PCR

Total mRNA was isolated from snap frozen whole liver tissue with the peqGOLD RNAPure Kit (Peqlab, Erlangen, Germany). cDNA was synthesized out of 500 ng mRNA after DNAse I digestion with the DNAse I Kit (Invitrogen, Karlsruhe, Germany). After reverse transcription with the Omniscripts reverse-transcription Kit (Qiagen, Hilden, Germany) Real-Time PCR was performed to measure cDNA expression of specific genes of interest. For this purpose the SybrGreen tqPCR Supermix (Invitrogen, Karlsruhe, Germany) was used. GAPDH expression was used as housekeeping gene. (Primer sequences: GAPDH forward: TGT TGA AGT CAC AGG AGA CAA CCT, reverse: AC CTG CCA AGT ATG ATG ACA TCA; TNFα forward: AGC ACA GAA AGC ATG ATC CG, reverse: CCC GAA GTT CAG TAG ACA GAA GAG; CCL2 forward: CCT CAT CCT CCA GCA TGA AGG TCT CTG C, reverse: GGT GGA GTC AGG CTT CAA GGC TTC GG, IL-2 forward: CCT GAG CAG GAT GGA GAA TTA CA, reverse: TCC AGA ACA TGC CGC AGA G; Granzyme B forward: TCG ACC CTA CAT GGC CTT AC, reverse: TGG GGA ATG CAT TTT ACC AT; α-SMA forward: ATG AAG CCC AGA GCA AGA GA, reverse: ATG TCG TCC AGT TGG TGA T; Col1A1 forward: GCT ACT ACC GGG CCG ATG ATG C, reverse: CCT TCG GGG CTG CGG ATG TTC).

### Western Blot Analysis

Whole liver tissue was lysed with ice cold lysis buffer for SDS–PAGE (1 M NaCl, 0.01 M EGTA, 0.5 M EDTA, 1 M NaH_2_PO_4_, 1 M Tris, 1 M NaF, 10× Triton, 0.1 M PMSF in EtOH, 1 mM Na_3_VO_4_). The protein lysat was denaturated at 95°C for 5 min in double-strength sodium dodecyl sulfate sample buffer with dithiothreitol before resolving in 10%% SDS–PAGE. Primary antibody incubation was performed with α-SMA (ab 32575, Abcam, Cambridge, United Kingdom) and β-Actin (MA1-744, Thermofisher, Waltham, Boston, MA, United States) antibodies. For detection secondary HRP-linked antibodies against rabbit IgG (7074S, Cell signaling, Frankfurt, Germany) or mouse IgG (sc-2005, Santa Cruz, Heidelberg, Germany) were used. To visualize the antigen-antibody complex the ECL Chemiluminescence Kit (GE Healthcare, Buckinghamshire, United Kingdom) was used.

### Immunofluorescent Staining

Snap frozen liver tissue in OCT was cut in 5 μm sections. After 30 min air-dry fixation was performed in 4% ice-cold paraformaldehyde. Primary antibody incubation (CD11b, 1:100, BD Pharmingen, cat. no.: 550282; PD1, 1:100, Life Technologies, cat. no.: PA520350) was performed for 1 h in 1% mouse serum with 0.02% sodium acetat in PBS. Secondary antibody detection (AlexaFluor 488-conjugated (CD11b) or AlexaFluor 594-conjugated (PD1) Molecular Probes, Boston, MA, United States) was performed for 1 h at room temperature. Nuclei were counterstained with DAPI (Vector Laboratories/Axxora, Lörrach, Germany).

### Extraction Procedure to Obtain Intrahepatic Lymphocytes (IHL) and Peripheral Blood Mononuclear Cells

Blood was collected and stored in EDTA tubes and treated with red blood cell lysing buffer from BD Biosciences (BD Pharm Lyse^TM^, cat. no.: 5555899) following the producer’s manual and stored on ice for subsequent FACS staining. To obtain IHL, we perfused the liver with PBS, detached the liver and removed the gall bladder. The left liver lobe was placed in HBSS (PAN Biotech, cat. no.: P04-34500) on ice, minced with scissors and digested with 0.2% collagenase type 4 (Worthington, cat. no.: 4188CL94) for 30 min at 37°C. Collagenase was stopped with flow buffer (PBS/0.5% BSA/2 mM EDTA) and tissue was further homogenized with a syringe and cannula and filtered through a 70 μm cell strainer (Falcon cell strainer 70 μm, Ref. no.: 352350) to gain single cell suspension. After a washing step, the liver cell pellet was dispensed in 8 ml 35% Percoll (GE Healthcare Life Sciences, cat. no.: 17-0891-01) and spun at 700 G for 30 min without brake at room temperature. The lowermost layer was lysed with lysis buffer to get again rid of the red blood cells. After a washing step, cells were distributed among the different FACS tubes.

### Flow Cytometry

Human intrahepatic leukocytes of 5 control and 5 NASH patients (Supplementary Table 2) were stained with surface antibodies for 30 min at 4°C in the dark (CD3-BV510 BD Biosciences, clone HIT3a, cat. no.: 564713; CD4-APC-Cy7 BD Biosciences, clone Leu3a, cat. no.: 566319; CD8-AlexaFluor700 BD Biosciences, clone RPA-T8, cat. no.: 561453; PD1-PE-Cy7 BD Biosciences, clone EH12.1, cat. no.: 561272; 2B4-PerCP-Cy5.5 Biolegend, clone C1.7, cat. no.: 329515; CD14-VioGreen Miltenyi Biotec, clone TÜK4, cat. no.: 130-098-061; CD68-PE-eFluor610 Thermofisher, clone Y1/82A, cat. no.: 61-0689-42). After staining and washing samples were analyzed at the BD Fortessa and quantified with the FlowJo^®^ software.

Mouse peripheral blood mononuclear cells and intrahepatic leukocytes were stained with surface antibodies for 30 min at 4°C in the dark (CD45-APC Cy7 BD Biosciences, clone 30F11, cat. no.: 557659; CD3-PECy7 eBioscience, clone 145-2C11, cat. no.: 25-0031-82; CD4-FITC eBioscience, clone GK1.5, cat. no.: 11-0041-85; CD4-APC eBioscience, clone GK1.5, cat. no.: 17-0041-82; CD44-APC; CD8-FITC eBioscience, clone 53-6.7, cat. no.: 11-0081-85; CD8-PerCPCy5.5 BD Biosciences, cat. no.: 551162; NK1.1-PE eBioscience, clone PK136, cat. no.: 12-5941-82; CD11b-PE eBioscience, clone M1/70, cat. no.: 12-0112-82; F4/80-APC eBioscience, clone BM8, cat. no.: 17-4801-82; Ly6C-PerCPCy5.5 BD Biosciences, clone AL-21, cat. no.: 560525; Ly6G-FITC BD Biosciences, clone 1A8, cat. no.: 551460; PD1-PerCPCy5.5 Biolegend, clone 29F.1A12, cat. no.: 135208; 2B4-PE eBioscience, clone eBio244F4, cat. no.: 12-2441-82; CD62L-PE Cy7 eBioscience, clone MEL-14, cat. no.: 25-0621-82; CD107a-APC BD Biosciences, clone 1D4B, cat. no.: 560646; CD25-PE eBioscience, clone PC61.5, cat. no.: 12-0251-82; CD69-PE eBioscience, clone H1.2F3, cat. no.: 12-0691-82; CD69-PerCPCy5.5 eBioscience, clone H1.2F3, cat. no.: 45-0691-82). After two washes with PBS, cells were ready for FACS analysis. The viability dye Hoechst (BD Biosciences, cat. no.: 340487) and counting beads (BD Biosciences cat. no.: 340487) were added to the samples shortly before measurement. Flow cytometric analysis was performed on a BD Canto II and analysis was realized using FlowJo^®^ software. Gating thresholds for the different surface antigens were determined by fluorescence minus one controls.

### Detection of De-Granulating CD8 T Cells by CD107a Assay

*In vivo*, intracellular cytokines are difficult to measure because of low concentrations and the impossibility to use protein secretion inhibitors like Brefeldin A or monensin. Therefore, we decided to detect cytotoxicity of activated CD8^+^ T cells by a flow cytometric assay for CD107a. De-granulating CD8 T cells are identified by surface expression of CD107a, also called lysosomal associated membrane protein, located in lysosomal granule membranes. In the course of CD8 T cell activation, lysosomal membranes fuse with the external plasma membrane for exocytosis of cytotoxic cytokines and the marker CD107a can transiently be detected at the cell surface. As we intended to detect *in vivo* activation of CD8 T and NK cells, we could not use Monensin to inhibit re-internalization and degradation of CD107-antibody complexes by endocytosis. Therefore, the measured percentages of activated cells reflect a snap shot of activation at one single time point.

### Multiplexed Immunofluorescence and Multispectral Image Analysis

Liver tissue sections were heated for 1 h at 60°C, immersed in 100% xylene for deparaffinization and rehydrated with a series of graded ethanol. After fixation in 10% neutral-buffered formalin for 20 min, antigen retrieval was performed using AR6 buffer (PerkinElmer) for 20 min in a 98°C water bath. Blocking was performed for 30 min with 10% donkey serum (Abcam) followed by 1 h incubation with the first primary antibody for PD1 (Life Technologies, cat. no.: PA520350) in a humidified chamber at room temperature. For detection the anti-rabbit secondary antibody (Cell Signaling Technology, cat. no.: 7074S) was used. Visualization was accomplished using the TSA Plus Cyanine 5 Kit (PerkinElmer), after which the antigen retrieval step was repeated. In a serial fashion, the slides were then incubated with the primary antibody for CD107a (Biozol, cat. no.: bs-1970R), followed by detection using anti-rabbit (Cell Signaling Technology, cat. no.: 7074S) after which CD107a was visualized using TSA Plus Cyanine 3 Kit (PerkinElmer). The slides were again placed in AR6 buffer and subjected to heat-induced antibody complex removal. Afterward the sections were incubated with the primary antibody against CD4 (Biozol, cat. no.: bs-0647R) for 1 h in a humidified chamber at room temperature, followed by detection using the anti-rabbit (Cell Signaling Technology, cat. no.: 7074S). Visualization of CD4 was accomplished using the TSA Plus Fluorescein Kit (PerkinElmer). After another incubation in AR6 buffer with heat induction for antibody complex removal, the sections were incubated with the last primary antibody CD8 (Biozol, cat. no.: bs-0648R), for 1 h in a humidified chamber at room temperature, followed by detection using the anti-rabbit (Cell Signaling Technology, cat. no.: 7074S). Visualization of CD8 was accomplished using the TSA Plus Cyanine 5.5 Kit (PerkinElmer). Counterstaining of cell nuclei was performed using Spectral DAPI (PerkinElmer) and the sections were embedded with Mowiol 4-88 (Roth).

For analysis whole multiplex stained liver sections were automatically scanned with the Vectra 3.0 Automated Quantitative Pathology Imaging System (PerkinElmer) with a 10× objective for the whole slide overview scan and 20× objective for multispectral images. The system requires individual examples of each fluorophore emission spectrum as well as a representative autofluorescence spectrum of an unstained sample to establish a spectral library that enables the spectral information to be reliably unmixed and quantified. The microscope captures fluorescent spectra with 20 nm wavelength intervals in a range from 420 to 720 nm and combines these captures into a single image stack, while retaining the spectral signature of the used fluorophores. Image files were then analyzed with the inForm 2.4.2 software (PerkinElmer), which employs a pattern recognition learning algorithm supporting a tissue and cell classifier tool. This was trained to segment and phenotype the individual stained cells and generated a pixel based intensity report for all antibodies used. Supplementary Figure 6 shows the training process applied for the individual analysis. After giving the system several examples for cell segmentation, the computer implemented an automated phenotyping for the whole image which was controlled by the scientist. As a last step, the computational calculation for cell types expressing the stained markers was performed. Based on the mean fluorescent intensity, a positivity threshold for each cell type was determined for scoring of each marker.

### Statistical Analysis

Calculations of two groups were performed with the Students unpaired *t*-test and the results were described as mean ± standard deviation. When comparing more than two groups, ANOVA testing with Turkey’s multiple comparison post-test was performed. At least five animals per group were analyzed. Outliers were excluded, when Grubb’s test calculated on graph pad homepage^1^ allowed their deletion. All the statistical tests were performed using Graph-Pad Prism^®^ software. A *p*-value of <0.05 was defined as significant (^∗^*p* < 0.05, ^∗∗^*p* < 0.01, and ^∗∗∗^*p* < 0.001).

## Results

### Feeding a WD for 24 Weeks Is an Appropriate Model to Reproduce Steatohepatitis and Metabolic Syndrome in Mice

**FIGURE 1:**
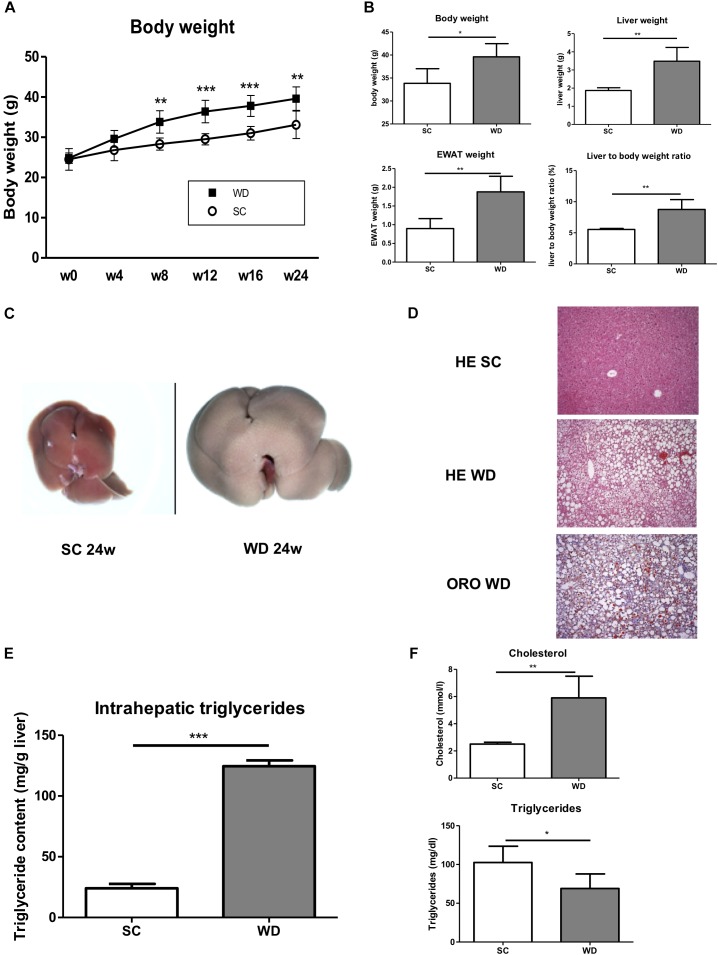
Metabolic phenotype of mice after feeding a WD for 24 weeks. **(A)** Body weight curve of male wild-type BL/6J mice fed SC or WD for 24 weeks. (*n* = 5, ^∗∗^*p* < 0.01 and ^∗∗∗^*p* < 0.001). **(B)** Body weight, liver weight, weight of epididymal white adipose tissue and liver to body ratio after feeding SC or WD for 24 weeks. (*n* = 5), (^∗^*p* < 0.05 and ^∗∗^*p* < 0.01) **(C)** Macroscopic aspects of livers from SC and WD fed mice **(D)** Representative H&E and Oil red O stained liver sections. **(E)** Intrahepatic triglyceride content after 24 weeks of SC and WD feeding. (*n* = 5, ^∗∗∗^*p* < 0.001). **(F)** Serum cholesterol and triglyceride levels after 24 weeks of SC or WD feeding. (*n* = 5, ^∗^*p* < 0.05 and ^∗∗^*p* < 0.01).

**FIGURE 2:**
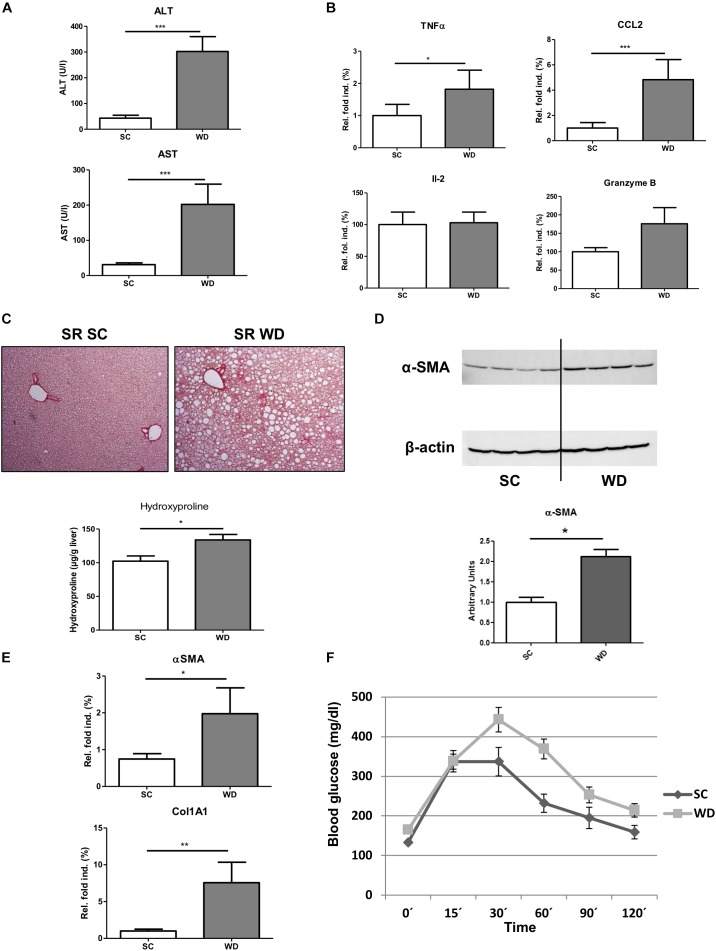
Stronger pro-inflammatory immune response, fibrosis development and diabetic phenotype in WD fed mice. **(A)** ALT and AST serum levels in mice fed a SC or WD for 24 weeks. (*n* = 5, ^∗∗∗^*p* < 0.001) **(B)** Hepatic gene expression of pro-inflammatory cytokines (tumor necrosis factor α, chemokine ligand 2, interleukin-2 and granzyme B) in mice fed SC or WD for 24 weeks. Values are expressed as relative fold induction over the mean values obtained for SC-fed animals. (*n* = 5, ^∗^*p* < 0.05 and ^∗∗∗^*p* < 0.001) **(C)** Sirius Red-stained liver sections and intrahepatic hydroxyproline levels of SC and WD fed mice. (*n* = 5, ^∗^*p* < 0.05) **(D)** Western blot analysis with quantitative evaluation for α-SMA in whole liver homogenates of SC and WD fed mice. (*n* = 4, ^∗^*p* < 0.05) **(E)** mRNA expression levels of α-SMA and Col1A1. Whole liver homogenates of chow and WD fed mice were analyzed via real-time PCR. The quantification is expressed as relative fold induction over the mean values obtained for SC fed mice. (*n* = 5, ^∗^*p* < 0.05 and ^∗∗^*p* < 0.01) **(F)** Mice fed SC or WD for 24 weeks showed an impaired glucose tolerance by stronger increase of blood glucose after intraperitoneal glucose administration compared to equally treated chow diet fed mice. (*n* = 5, ^∗^*p* < 0.05).

Mice fed with a WD for 24 weeks showed a significant gain in body weight, liver weight and weight of epididymal white adipose tissue (Figure 1A,B). The weight gain of the liver was even more pronounced than the general weight gain, as shown by an augmented liver body weight ratio (Figure 1B). Macroscopic appearance of fatty liver already differed clearly from healthy liver by a much larger size and a brighter color (Figure 1C). HE staining and Oil red O staining confirmed lipid accumulation in livers of WD fed mice (Figure 1D). In addition, measuring intrahepatic triglycerides revealed fivefold elevated hepatic triglyceride content in WD fed mice than SC diet fed controls (Figure 1E). Additionally, serum cholesterol levels were increased in WD mice, whereas serum triglyceride serum levels decreased (Figure 1F). The last-named observation was also shown by other investigators (Clapper et al., 2013) and seems to be an undesirable effect of this otherwise good performing NASH diet. In addition, it has to be mentioned, that mice were not set fasting for measurement of serum triglycerides. Serum ALT and AST level confirmed hepatic inflammation (Figure 2A), which was also reflected by elevated intrahepatic pro-inflammatory cytokines TNF-α and CCL2 (Figure 2B). Interestingly, markers of T cell activation like interleukin-2 and granzyme B remained stable (Figure 2B), potentially reflecting a mechanism of T cell inhibition in NASH. In contrast to many other dietary NASH models, this WD was also able to produce steatosis-induced fibrosis as shown by Sirius Red staining and hydroxyproline assay (Figure 2C), α-SMA expression (Figure 2D,E), and Col1A1 transcription products (Figure 2E). Furthermore, this WD created signs of diabetes mellitus as demonstrated by elevated fasting glucose serum levels and pathological glucose tolerance after weight-adapted intraperitoneal glucose injection (Figure 2F).

### Inhibitory T Cell Receptors PD1 and 2B4 Play a Pivotal Role in the Liver

**FIGURE 3:**
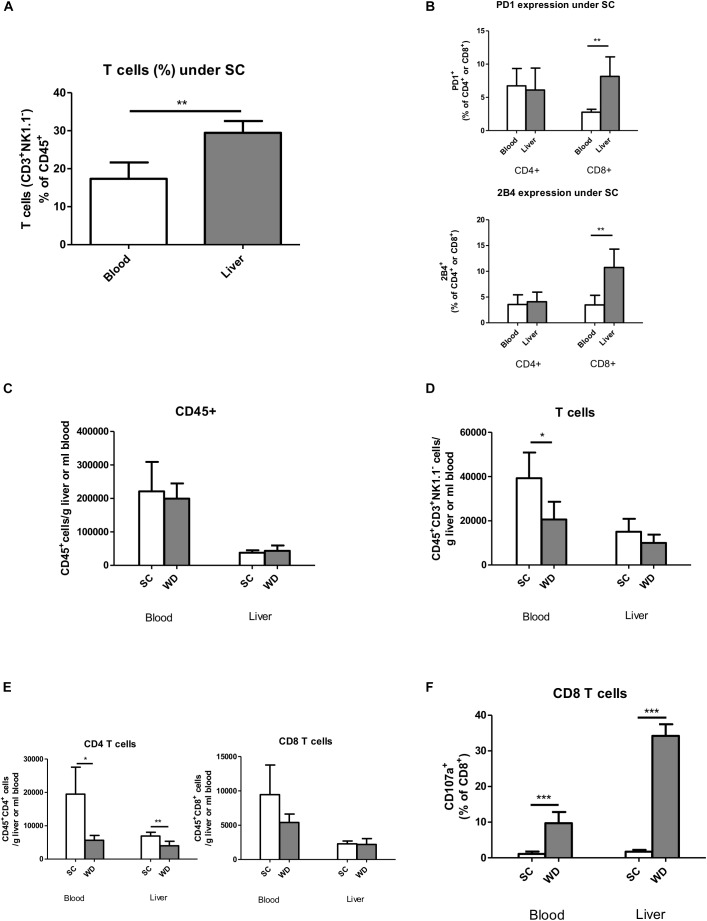
T cell characterization by flow cytometry in the blood and livers of chow and WD fed mice. **(A)** T cells (CD45+Hoechst-CD3+NK1.1-) in the blood and liver after SC feeding. (*n* = 5, ^∗∗^*p* < 0.01) **(B)** Basic PD1 and 2B4 expression in blood and liver CD45+CD4+ and CD45+CD8+ T cells under SC. (*n* = 5, ^∗∗^*p* < 0.01) **(C)** Absolute leukocyte cell numbers in blood and liver after SC and WD feeding. (*n* = 5). **(D)** Absolute T cell numbers in blood and liver after feeding a SC or WD for 24 weeks. (*n* = 5, ^∗^*p* < 0.05) **(E)** Absolute CD4+ and CD8+ T cell numbers after SC feeding and after a metabolic challenge by a 24 week fed WD. (*n* = 5, ^∗^*p* < 0.05 and ^∗∗^*p* < 0.01) **(F)** Percentage of CD107a positive CD8+ T cells in blood and liver tissue after administration of a SC or WD for 24 weeks. (*n* = 5, ^∗∗∗^*p* < 0.001).

Under SC conditions, liver T cells (Hoechst^-^, CD45^+^, CD3^+^, and NK1.1^-^) form a higher percentage of total leukocytes (Hoechst^-,^ CD45^+^; 29.42%, SD 3.15%) than blood T cells (18.58%, SD 3.85%; *p* = 0.005) (Figure 3A) (for gating strategies see Supplementary Figures 1–3). Therefore, functional regulation of T cells by activating and inhibitory receptors could be especially important for immunological balance in the liver. Under normal alimentary conditions, the inhibitory receptors PD1 and 2B4 show higher expression on hepatic than blood CD8 T cells (8.2 and 10.8% vs. 2.8 and 3.5%, respectively) (Figure 3B). Therefore, their regulation under metabolic burden could be especially important to control CD8 T cell activity in NASH liver. In contrast, hepatic and blood CD4 T cells did not differ in expression levels of these inhibitory receptors in SC fed mice (Figure 3B).

### Fatty Livers Show a Selective CD4 T Cell Loss

Feeding a WD for 24 weeks resulted in unchanged total intrahepatic leucocyte numbers (CD45^+^ Hoechst^-^; SC: 37,696 cells/g liver, SD 7,478 cells/g liver; WD: 43,433 cells/g liver, SD 15,957 cells/g liver) (Figure 3C) and unchanged hepatic T cells numbers (CD45^+^ Hoechst^-^, CD3^+^; SC: 15,043 cells/g liver, SD 5,822 cells/g liver; WD: 9,999 cells/g liver, SD 3,710 cells/g liver) (Figure 3D) with diminished CD4 T cells (CD45^+^Hoechst^-^CD4^+^; SC: 6,922 cells/g liver, SD 1,106 cells/g liver; WD: 3,976 cells/g liver, SD 1,370 cells/g liver; *p* = 0.0057) (Figure 3E) and stable CD8 T cell numbers (CD45^+^Hoechst^-^CD8^+^; SC: 2,276 cells/g liver, SD 417 cells/g liver; WD: 2,193 cells/g liver, SD 851 cells/g liver) (Figure 3E). At the same time, myeloid cells increased intrahepatically under WD treatment (CD45^+^Hoechst^-^Ly6G^-^CD11b^+^; SC: 22,718 cells/g liver, SD 4,075 cells/g liver; WD: 42,048 cells/g liver, SD 13,272 cells/g liver; *p* = 0.0144) (Supplementary Figure 4A) with stable numbers of monocytes (CD45^+^Hoechst^-^Ly6G^-^CD11b^++^F4/80^+^; SC: 5,117 cells/g liver, SD 1,178 cells/g liver; WD: 7,227 cells/g liver, SD 2.794 cells/g liver) and activated monocytes (CD45^+^Hoechst^-^Ly6G^-^CD11b^++^F4/80^+^Ly6C^+^; SC: 1,350 cells/g liver, SD 691 cells/g liver; WD 2,032 cells/g liver, SD 1,066 cells/g liver) (Supplementary Figure 4B) but increased numbers of liver macrophages (Kupffer cells) (CD45^+^Hoechst^-^Ly6G^-^CD11b^+^F4/80^++^; SC: 274 cells/g liver, SD 84 cells/g liver; WD 3,470 cells/g liver, SD 1,181 cells/g liver; *p* = 0.0003) (Supplementary Figure 4C) under metabolic surcharge. The hepatic neutrophil cell number was also markedly increased under WD (CD45^+^Hoechst^-^Ly6G^+^; SC: 397 cells/g liver, SD 356 cells/g liver; WD: 4,019 cells/g liver, SD 2,282 cells/g liver; *p* = 0.0080) (Supplementary Figure 4D), whereas hepatic NK cell number (CD45^+^Hoechst^-^CD3^-^NK1.1^+^; SC: 3,999 cells/g liver, SD 1,495 cells/g liver; WD 4,610 cells/g liver, SD 1,018 cells/g liver) (Supplementary Figure 4E) remained stable and NKT cell numbers (CD45^+^Hoechst^-^CD3^+^NK1.1^+^CD8^+^; SC: 179 cells/g liver, SD 36 cells/g liver; WD: 68 cells/g liver, SD 26 cells/g liver; *p* = 0.0021) (Supplementary Figure 4F) were even reduced under WD.

### CD8 T Cells Show Increased Degranulation Capacity in NASH Liver

In NASH liver, CD8 T cell and NK cell numbers remained unchanged (Figure 3E and Supplementary Figure 1E), but these cells showed a more activated phenotype as indicated by increased CD107a expression on their cell surface (Figure 3F and Supplementary Figure 2B). This increased degranulation capacity under WD was more prominent in the liver for CD8 T cells and even liver specific for NK cells. It has to be mentioned, that – in contrast to *in vitro* studies, where CD107a expression is measured cumulatively for a stimulation period of 4–6 h – we were only able to measure CD107a expression of our *in vivo* stimulated CD8 T- and NK cells for a single time point. In *in vitro* experiments, however, the addition of monensin during the activation period prevents acidification and subsequent degradation of re-endocytosed CD107a-antibody complexes and therefore results in higher cell numbers of CD107a positive, de-granulating CD8 T and NK cells.

### In Steatohepatitis, Inhibitory Receptor 2B4 Is Upregulated on Hepatic CD4 and CD8 T Cells. Additional Upregulation of PD1 on CD8 T Cells Correlates With Lower Degranulation Capacity

**FIGURE 4:**
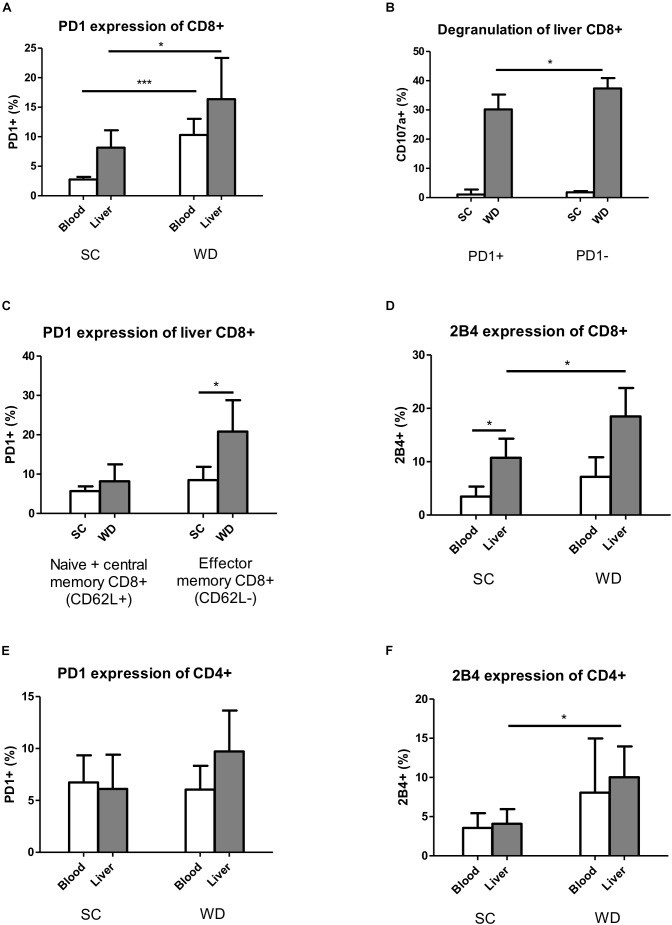
Expression of inhibitory T cell receptors PD1 and 2B4 increases on hepatic T cells under WD. **(A)** Blood and intrahepatic PD1 expressing CD8+ cells were analyzed by Flow Cytometry after SC and WD feeding for 24 weeks. (*n* = 5, ^∗^*p* < 0.05 and ^∗∗∗^*p* < 0.001). **(B)** Percentage of intrahepatic degranulating CD107a+ CD8 T cells of PD1+ and PD1- cells after SC or WD treatment for 24 weeks. (*n* = 5, ^∗^*p* < 0.05). **(C)** Upregulation of PD1 on intrahepatic CD8+ CD62L+ (Naïve + central memory CD8+ T cells) and CD8+ CD62L- (effector memory CD8+ T cells) T cells after SC or WD. (*n* = 5, ^∗^*p* < 0.05). **(D)** 2B4 expression on CD8+ T cells in blood and liver after SC or WD feeding for 24 weeks. (*n* = 5, ^∗^*p* < 0.05). **(E)** PD1 expression on blood and hepatic CD4+ T cells under SC or WD for 24 weeks. (*n* = 5). **(F)** Inhibitory receptor 2B4 expression blood and intrahepatic CD4 T cells under SC or WD. (*n* = 5, ^∗^*p* < 0.05).

As mentioned above, under normal nutrition conditions, the inhibitory receptor PD1 showed higher expression levels on hepatic than blood CD8 T cells. Feeding a WD led to an upregulation of PD1 on liver and blood CD8 T cells (Figure 4A). As CD8 T cell numbers were unchanged in liver, but by trend decreased in blood, this effect could be especially important for the integrity of the liver. Under WD, PD1 expressing CD8 T cells degranulate less than PD1 negative CD8 T cells, as shown by reduced CD107a expression levels (Figure 4B). The upregulation of PD1 was restricted to effector memory T cells, defined as CD62L negative cells (CD45^+^Hoechst^-^CD8^+^CD62L^-^) (Figure 4C), whereas naive and central memory CD8 T cells (CD45^+^Hoechst^-^CD8^+^CD62L^+^) showed unchanged low expression levels of PD1.

The second analyzed inhibitory T cell receptor 2B4 showed higher basic expression levels on liver than blood CD8 T cells and was upregulated on liver CD8 T cells upon WD challenge, but unchanged on blood CD8 T cells (Figure 4D). In contrast to CD8 T cells, CD4 T cells did not show any differences in the expression levels of PD1 and 2B4 between liver and blood under SC conditions (Figure 4E,F). Under metabolic challenge, the inhibitory receptor PD1 was not regulated on CD4 T cells neither in liver nor in blood (Figure 4E). Contrary to PD1, 2B4 was increased on hepatic CD4 T cells, but unchanged on blood CD4 T cells under high caloric diet (Figure 4F).

The results obtained for PD1 and CD107a co-expression on CD4^+^ and CD8^+^ T cells could further be confirmed using a multiplex staining approach on whole liver tissue sections (Supplementary Figure 5). This staining was quantified by a pattern recognition learning algorithm supporting a tissue and cell classifier (Supplementary Figure 6). Co-staining for CD11b and PD1 further showed that also myeloid lineage cells express PD1 (Supplementary Figure 7).

### Activating T Cell Receptors Are Especially Important on CD4 T Cells in Steatohepatitis

**FIGURE 5:**
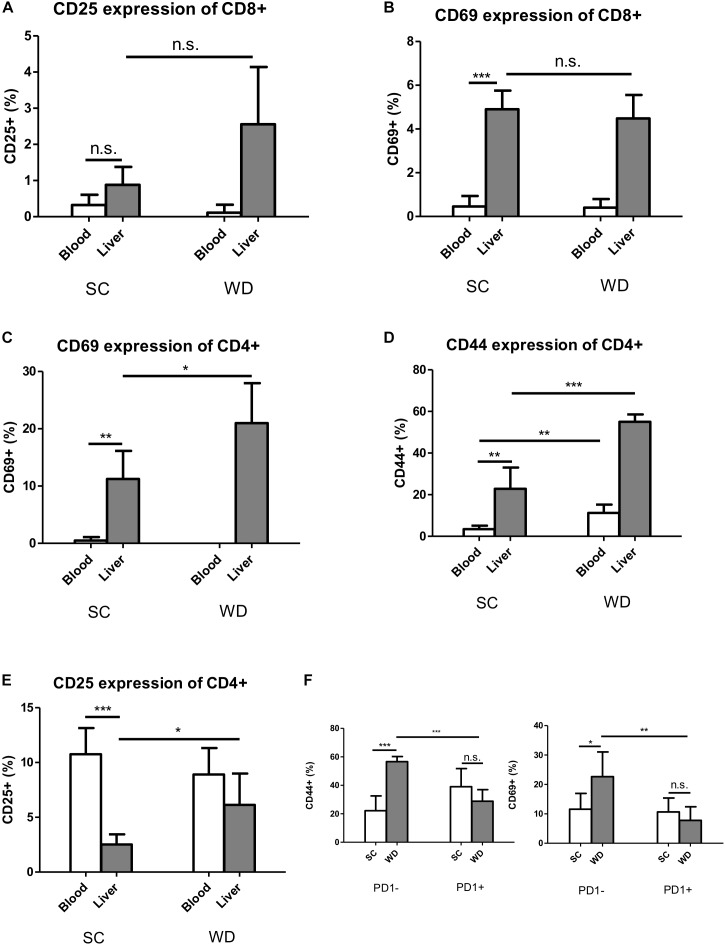
Activation markers CD69 and CD44 are increased under metabolic stress on hepatic CD4 T cells, but not CD8 T cells, while this activation is inhibited in PD1+ CD4 T cells. **(A)** CD25 expression on blood and liver CD8 T cells under SC or WD for 24 weeks. (*n* = 5). **(B)** CD69 expression on blood and on hepatic CD8 T cells after 24 weeks treatment with SC or WD. (*n* = 5), (^∗∗∗^*p* < 0.001). **(C)** CD69 expression on blood and hepatic CD4 T cells after 24 weeks SC and WD feeding. (*n* = 5, ^∗^*p* < 0.05). **(D)** CD44 expression on blood and intrahepatic CD4 T cells under SC or WD treatment for 24 weeks. (*n* = 5, ^∗∗^*p* < 0.01 and ^∗∗∗^*p* < 0.001). **(E)** CD25 expression on blood and hepatic CD4 T cells under SC or WD. (*n* = 5), (^∗^*p* < 0.05 and ^∗∗∗^*p* < 0.001). **(F)** CD44 and CD69 expression on PD1+ or PD1- CD4 T cells after SC or WD feeding. (*n* = 5), (^∗^*p* < 0.05; ^∗∗^*p* < 0.01; and ^∗∗∗^*p* < 0.001).

Similar to the inhibitory receptors PD1 and 2B4, the activation markers CD25 and CD69 were by trend higher expressed in liver than blood CD8 T cells under basic conditions (Figure 5A,B). In contrast, their expression levels remained unchanged on hepatic and blood CD8 T cells under metabolic burden (Figure 5A,B). The activation marker CD69 was only expressed on liver CD4 T cells and CD44 showed a higher expression level on hepatic CD4 T cells than blood CD4 T cells already under SC conditions, whereas CD25 showed higher basic expression on blood CD4 T cells (Figure 5C–E).

Under WD, CD69 and CD25 were upregulated on CD4 T cells liver-specifically (Figure 5C–E), whereas the activation marker CD44 was upregulated on both, liver and blood CD4 T cells. Albeit PD1 was not upregulated on liver CD4 T cells under metabolic stress, hepatic PD1 positive CD4 T cells showed reduced co-expression of CD69 and CD44 (Figure 5F), indicating that this cell population is inhibited by the PD1 molecule.

### In NASH Patients, PD1 Is Upregulated on CD8 T Cells and CD14/CD68 Monocytes. Additional the Inhibitory Receptor 2B4 Is Upregulated on Hepatic CD4 and CD8 T Cells but Not on CD14/CD68 Monocytes

**FIGURE 6:**
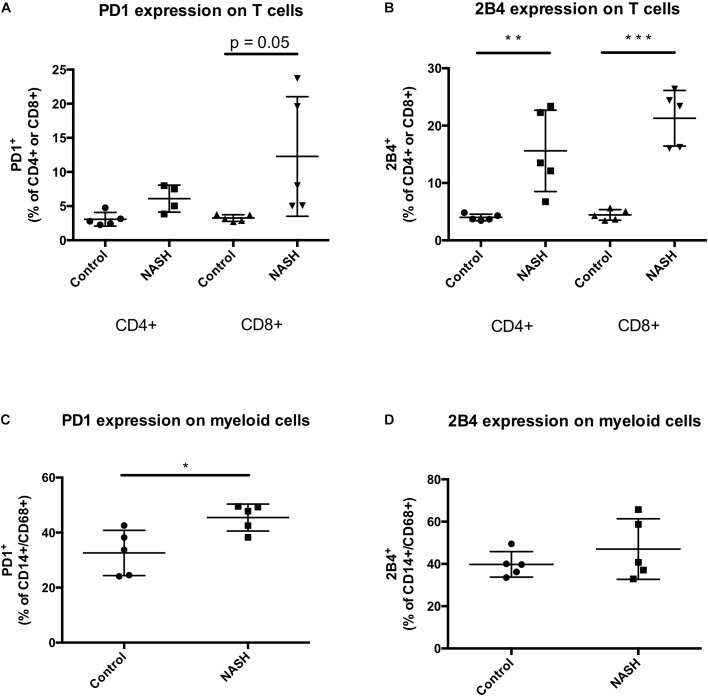
PD1+ and 2B4+ infiltrating cells are increased in NASH patients. **(A)** Intrahepatic PD1+ CD4 or CD8+ T cells and **(B)** 2B4+ CD4 or CD8+ T cells were analyzed by flow cytometry. Analysis include 5 control and 5 NASH patients. Intrahepatic cells were gated FSC/SSC, duplets were excluded, living cells, CD3+, PD1+/CD4+, PD1+/CD8+, 2B4+/CD4+, 2B4+/CD8+. Displayed is the percentage of recorded cells in liver biopsy samples. **(C)** Intrahepatic PD1+/CD14+/CD68+ and **(D)** 2B4+/CD14+/CD68+ monocytes were analyzed by flow cytometry. Analysis include 5 control and 5 NASH patients. Intrahepatic cells were gated FSC/SSC, duplets were excluded, living cells, CD14+/CD68+, PD1+ or 2B4+. Displayed is the percentage of recorded cells in liver biopsy samples. (^∗^*p* < 0.05; ^∗∗^*p* < 0.01; and ^∗∗∗^*p* < 0.001).

Non-alcoholic steatohepatitis patients showed the tendency to have higher intrahepatic levels of PD1 expressing CD8 T cells compared to control patients reflecting the results obtained in the mouse experiments (Figure 6A). The CD4 and CD8 T cell numbers expressing the inhibitory T cell receptor 2B4 were significantly increased in livers of patients with NASH compared to control (Figure 6B). In NASH patients the amount of intrahepatic CD14/CD68 monocytes was significantly higher than in control patient livers (Figure 6C). Contrary to PD1 expressing monocytes, 2B4 was not differentially expressed on CD14/CD68 monocytes in human NASH livers (Figure 6D).

In conclusion, under WD the CD8 T cell number in liver remained stable but showed a more inhibited phenotype with high expression of inhibitory receptors PD1 and 2B4 and no alterations of the activating receptors CD25 and CD69. In contrast, hepatic CD4 T cell number decreased. The remaining CD4 T cells showed a liver-specific upregulation of only 2B4 with concomitant upregulation of the activating receptors CD44 and CD69. Thus, in fatty liver, the cytotoxic T cell response is functionally suppressed by overexpression of inhibitory T cell receptors, whereas the CD4 T cell response is limited by decreased cell numbers.

## Discussion

In our study, already under normal nutritional conditions, T cells turned out to represent a big fraction of intrahepatic leukocytes compared to the peripheral leukocyte composition. Furthermore, in healthy mouse livers, cytotoxic CD8 T cells expressed higher levels of the inhibitory T cell receptors PD1 and 2B4 (Figure 3), whereas CD4 T helper cells expressed higher proportions of the activating receptors CD44 and CD69 (Figure 6), both compared to their blood counterparts. The high hepatic T cell number and the liver specific distribution pattern of regulating T cell receptors motivated us to further analyze the regulation of inhibitory and activating T cell receptors in a mouse model of NASH and in human NASH patients.

At first glance, the administration of a WD for 24 weeks seemed to be accompanied by little T cell-mediated damage, as – in contrast to hepatic macrophages and neutrophils (Supplementary Figure 4) – CD8 T cell number remained unchanged and the CD4 T cell population was even reduced. Recently, a study confirmed a selective loss of CD4 T cells in NAFLD, which was ascribed to increased oxidative stress by linoleic acid (Ma et al., 2016). A shifting of the CD4/CD8 ratio in favor of CD8 could also be confirmed by this group in a HFD model and by other investigators, when applying a MCD model of NASH for 4 or 6 weeks, respectively (Henning et al., 2013; Kroy et al., 2014b).

However, examining for CD107a expression as a marker of CD8 T cell degranulation revealed, that CD8 T cells de-granulated more under metabolic surcharge and this effect was more pronounced in the liver (Figure 3) than in periphery. In a CD-HFD model of NASH, Wolf et al. could also describe metabolically activated CD8 T cells, monitored by CD44 and CD69 upregulation (Wolf et al., 2014), underlining that CD8 T cells could play a pro-inflammatory role in metabolic liver disease. In addition, they could show that a genetic knockout of CD8 T cells (Rag1^-/-^, β2m^-/-^ mice) and antibody mediated CD8 T cell depletion prevented from steatohepatitis (Wolf et al., 2014). Also, in other manifestation forms of the metabolic syndrome, such as diabetes and obesity, CD4 and CD8 T cells seem to play a disease worsening role. This is suggested by the fact that genetic CD4 and CD8 T cell deficiency (TCRβ^-/-^) in diet induced obese mice resulted in attenuated adipose tissue and skeletal muscle inflammation and a protection from hyperglycemia and insulin resistance (Khan et al., 2014). Another group showed that blocking T cell co-stimulation by weekly injection of anti-CD40L antibody in an obesogenic diet reduced adipose tissue inflammation, weight gain and improved insulin resistance by trend (Montes et al., 2013). Taken together, these preceding studies suggest a detrimental role for CD8 T cells in NASH development. However, as they applied non-liver-specific CD8 T cell knock out models, an extrahepatic CD8 T cell inhibition resulting in a modified microbiome or their inhibition in fatty tissue could play the critical role in NASH suppression. Nevertheless, these studies prompted us to further examine a suspected T cell mediated liver damage and the role of inhibitory T cell receptors in NASH.

To elucidate whether regulating T cell receptors could actually be important for hepatic inflammation, we first analyzed the expression patterns of the inhibiting T cell receptors PD1 and 2B4 under normal chow conditions in livers and blood. It turned out, that the inhibitory receptors PD1 and 2B4 were expressed to a higher level on hepatic than blood CD8 T cells (Figure 3). In contrast, the CD4 T cell compartment did not show any differences in PD1 or 2B4 expression between liver and blood T cells (Figure 3). Also, analysis of human NASH samples and in human studies, intrahepatic T cells show in general higher expression levels of the receptors PD1 and 2B4, independent of the underlying liver disease (Golden-Mason et al., 2007; Radziewicz et al., 2007; Kroy et al., 2014a; Figure 6). This higher basic expression could even be seen in healthy individuals and therefore probably represents a physiological reaction to the liver micro-environment that allows self-tolerance and is not a pathological consequence. It could be part of the liver specific immune-tolerance required for this organ getting in contact with numerous antigen structures via portal vein flow. Additional to cytotoxic T cells, in human specimen also CD4 T cells showed higher expression of the inhibitory receptors PD1 and 2B4 on liver versus blood CD4 T cells. However, also in humans there was a more pronounced difference in PD1 and 2B4 expression between liver and blood T cells within the CD8 T cell population (Kroy et al., 2014a).

Next, we exposed mice to a metabolic surcharge by administration of a WD for 24 weeks and analyzed the expression profile of inhibitory T cell receptors on hepatic and peripheral T cells. Nutritional excess resulted in an upregulation of the inhibitory receptor PD1 on liver and blood CD8 T cells (Figure 4). As CD8 T cell numbers were unchanged in liver, but by trend decreased in blood, this regulation could be especially important for self-tolerance in the liver. The upregulation of PD1 could further be localized to the effector memory CD8 T cell compartment, that subsequently showed reduced degranulation properties (Figure 4). An effector memory T cell-restricted upregulation of PD1 was also described for HCV/HIV-coinfection, where PD1 upregulation on CD8 T cells resulted in a diminished proliferation rate, reduced CD8 T cell degranulation measured by CD107a-expression and reduced interleukin-2 and interferon-y (INF-γ) levels (Saha et al., 2013). In fact, our study did not demonstrate reduced interleukin-2 concentration, but this could be due to different methodical approaches. We measured interleukin-2 on transcriptional level (mRNA) in whole liver homogenates, whereas Saha et al. performed isolation of effector memory CD8 T cells and FACS analysis of interleukin-2 after an *in vitro* stimulation phase while blocking cellular interleukin secretion with brefeldin A.

The significance of PD1 mediated inhibition of hepatic CD8 T cells in context of NASH is also stressed by a recent publication by Shalapour et al., that described increased intrahepatic programmed death ligand 1 (PD-L1) concentrations in humans and mice with NAFLD (Shalapour et al., 2017).

In contrast to CD8 T cells, CD4 T cells did not upregulate PD1 neither in liver nor in blood when fed a WD (Figure 4). Of note, total number of hepatic CD4 T cells were already strongly diminished under WD, therefore suppression by PD1 could be of minor relevance. Nevertheless, those hepatic CD4 T cells expressing PD1 showed an inhibited phenotype with reduced CD44 and CD69 expression (Figure 5). A specific inhibition of intrahepatic CD8 T cells but not CD4 T cells by upregulation of PD1 was also observed in human chronic HCV infection (Kroy et al., 2014a).

Similar to PD1, the inhibitory receptor 2B4, also called natural killer cell receptor CD244, was expressed in a higher extend on hepatic than peripheral CD8 T cells already under normal nutritional conditions. In contrast, CD4 T cells showed a weak expression level in liver as well as periphery. This finding reproduces in part our results for healthy humans, where 2B4 expression was generally higher in liver than blood and higher for CD8^+^ than CD4^+^ T cells (Kroy et al., 2014a). As a result, in humans 2B4 expression was most important in liver CD8^+^ cells as seen in our mouse experiment for chow diet fed mice.

WD produced a liver-specific upregulation of 2B4 on CD4 and CD8 T cells. In addition, 2B4 expression was higher in the hepatic CD8^+^ T cell population than the hepatic CD4 T cell population (Figure 4).

Increased expression of 2B4 on hepatic CD8 T cells was also described for other liver diseases such as chronic hepatitis B and chronic hepatitis C and their blockade yielded in an increased CD8 T cell toxicity with proliferation and the secretion of inflammatory cytokines like IFN-γ and TNFα (Raziorrouh et al., 2010; Owusu et al., 2015).

Taken together, our study revealed a pivotal role of the inhibitory T cell receptors PD1 and 2B4 for the CD8 T cell subset in liver. A higher expression of these inhibitory T cell receptors in CD8 T cells could already been seen under SC. In liver steatosis, PD1 and 2B4 expression were higher in the CD8 T cell population than the CD4 T cell population. The predominant role of PD1 and 2B4 for suppressing CD8 T cells in NASH liver resembles their expression pattern in human chronic hepatitis C infection (Kroy et al., 2014b). In this preceding study, 90% of intrahepatic CD8 T cells showed positive for 2B4 and 70% were positive for PD1, whereas only 30% of the CD4 T cell population were stained positive for 2B4 and 50% expressed PD1. Thus, in liver steatosis, CD8 T cell response is functionally suppressed by overexpression of inhibitory T cell receptors PD1 and 2B4 whereas CD4 T cell response is limited by decreased cell numbers.

Further studies have to clarify the mechanistic insights how WD leads to upregulation of these inhibitory T cell receptors on hepatic T cells. One possible explanation could be an intestinal bacterial overgrowth or a modified intestinal flora together with an increased intestinal permeability in NASH (Brun et al., 2007; Miele et al., 2009). The presence of a modified bacterial flora could induce PD1-upregulation, as described for type 1 diabetes mellitus, where a *Salmonella typhimurium* infection could protect from disease manifestation by upregulation of the PD1/PD-L1 axis (Newland et al., 2011). A leaky gut barrier results in increased LPS serum levels in NASH patients as well as murine NASH models and results in a TLR4 activation (Rivera et al., 2007; Farhadi et al., 2008; Csak et al., 2011). This increased LPS level under HFD could trigger upregulation of PD1 on T cells via TLR4 activation on monocytes. This mechanism was described for PD1 induction in human acute alcoholic hepatitis (Markwick et al., 2015) and fits well to NASH pathogenesis described above. Besides TLR4, other receptors potentially involved in PD1 and 2B4 upregulation could be TLR2 and TLR9, as bacterial DNA as a TLR9 ligand was elevated in blood of HFD fed mice (Miura et al., 2010) and free fatty acids as well as denatured host DNA could activate these two receptors in the course of NASH development (Senn, 2006; Watanabe et al., 2007). 2B4 is upregulated on human monocytes after liver X receptor (LXR) activation (Rébé et al., 2012), therefore its upregulation on hepatic T cells could also involve this nuclear receptor (Rébé et al., 2012).

In addition, it could be an intriguing question, if the endogenous immunosuppression by upregulated inhibitory T cell receptors in fatty liver exceeds the needed immunosuppression for lipid-induced inflammation and goes along with for example a decreased rejection rate in the context of liver transplantation. Such an effect could be detected in chronic viral hepatitis, where 2B4 and PD1-mediated T cell exhaustion protected patients from rejection (Bohne et al., 2014). In the same way, the upregulation of PD1 and 2B4 on hepatic T cells in NASH livers could protect NASH patients from liver transplant rejection.

Our multiplexed immunofluorescence analysis revealed an increased expression of PD1 in fatty liver, which was not completely restricted to the CD4 and CD8 T cell populations (Supplementary Figures 6, 7). As PD1 is also expressed on myeloid cells and especially macrophages were shown to increase dramatically in NASH livers, further studies could elucidate the functional role of PD1 expression on other infiltrating immune cells especially myeloid cells in fatty liver.

Concerning activating T cell receptors, metabolic burden elicited an increased expression of CD44, CD69 and the Il-2 receptor component CD25 on CD4 T cells. In contrast, no changes in the expression level of the stimulating receptors CD69 and CD25 on CD8 T cells could be detected.

The activating T cell receptor CD44 has also been confirmed to play a leading role in metabolic diseases. CD44 expression augments in liver and white adipose tissue in obese patients as well as rodents and correlates with liver steatosis and type 2 diabetes (Bertola et al., 2009; Kodama et al., 2012; Liu et al., 2015). Consequently, mice deficient in CD44 show a reduced hepatic steatosis, reduced inflammatory infiltrate in white adipose tissue and improved glucose tolerance (Kang et al., 2013). Importantly, treatment with anti-CD44 antibody improved insulin resistance, adipose inflammation, hepatic steatosis and weight gain in diet induced obese mice (Kodama et al., 2015), reflecting the potential impact of modulating T cell receptors on the outcome of metabolic diseases.

To sum up, in our mouse model of NASH and human NASH samples, upregulation of the inhibitory receptors PD1 on hepatic CD8 T cells and 2B4 on hepatic CD4 and CD8 T cells seems to limit T cell mediated liver damage. Therefore – contrary to cancer or chronic viral infections such as HBV, HCV, LCMV and HIV (Ye et al., 2015) – upregulation of these inhibitory T cell receptors could be beneficial in the context of metabolic liver disease. As a next step, the protective role of inhibitory T cell receptors in NASH development has to be confirmed in a dietary NASH model with PD1^-/-^ and 2B4^-/-^ mice. Future pharmacological strategies could apply PD1 and 2B4 agonists or induce their upregulation by an artificial non-harmful viral hepatic infection.

## Data Availability

All datasets generated for this study are included in the manuscript and/or the Supplementary Files.

## Ethics Statement

This study was carried out in accordance with the recommendations of the “Landesamt für Natur, Umwelt und Verbraucherschutz Nordrhein-Westfalen (LANUV)” and the Ethics Committee rules from liver explants or liver resection recruited at the RWTH Aachen University Hospital (local IRB permit number EK 166-12). The protocol was approved by the “Landesamt für Natur, Umwelt und Verbraucherschutz Nordrhein-Westfalen (LANUV)” and the Ethics Committee rules from liver explants or liver resection recruited at the RWTH Aachen University Hospital (local IRB permit number EK 166-12).

## Author Contributions

CH designed the study, acquired and analyzed the data, and drafted the manuscript. MB acquired and analyzed the data, critically revised the manuscript. TL was responsible for fundraising and critical revision of the manuscript. RW drafted and critically revised the manuscript. CT critically revised the manuscript and intellectual content. DK was responsible for the design and supervision of the study, fundraising, and drafting of the manuscript. HD designed and supervised the study, acquired the data, and drafted the manuscript. SE provided technical support.

## Conflict of Interest Statement

The authors declare that the research was conducted in the absence of any commercial or financial relationships that could be construed as a potential conflict of interest.

## Footnotes

1 http://graphpad.com/quickcalcs/Grubbs1.cfm

## Abbreviations

α-SMA: α-smooth muscle actin
ALT: alanine aminotransferase
ASH: alcoholic steatohepatitis
AST: aspartate aminotransferase
CCL2: CC chemokine ligand 2
CD107a: cluster of differentiation 107a
CD-HFD: choline deficient high fat diet
Col1A1: Collagen 1A1
GTT: glucose tolerance test
HBV: hepatitis B virus
HCV: hepatitis C virus
HFD: high fat diet
IHL: intrahepatic lymphocytes
IFN-γ: interferon-γ
LCMV: lymphocytic choriomeningitis virus
LPS: lipopolysaccharide
MCD: methionine choline deficient diet
NAFLD: Non-alcoholic fatty liver disease
NASH: Non-alcoholic steatohepatitis
PD1: programmed cell death protein 1
PD-L1: programmed cell death 1 ligand 1
SC: standard chow
TLR: toll like receptor
WD: Western diet
