# Supplementary material for: The Inhibitory T Cell Receptors PD1 and 2B4 Are Differentially Regulated on CD4 and CD8 T Cells in a Mouse Model of Non-alcoholic Steatohepatitis
Source: Front Pharmacol. 2019 Mar 13;10:244. doi: 10.3389/fphar.2019.00244 (PMC6436071; doi:10.3389/fphar.2019.00244)

Supplementary Tables and Figures

Supplementary Table 1: Composition of the fed HFD and SC diet.

Supplementary Table 2: Baseline patient characteristics used for FACS analysis.

Supplementary Figure 1: Gating strategy of immune cells.

Supplementary Figure 2: Antigen expression on $CD4^+$ T cells and corresponding fluorescence minus one controls.

Supplementary Figure 3: Antigen expression on $CD8^+$ T cells and corresponding fluorescence minus one controls.

Supplementary Figure 4: Modification of immune cell numbers after administration of a SC or a WD for 24 weeks. (A) Myeloid cells ($CD45^+Hoechst^-Ly6G^-CD11b^+$) in blood and liver after SC or WD administration for 24 weeks. (n=5), (* p<0.05). **(B)** Blood and intrahepatic monocyte ($CD45^+Hoechst^-Ly6G^-CD11b^{++}F4/80^+$) and activated monocyte numbers ($CD45^+Hoechst^-Ly6G^-CD11b^{++}F4/80^+Ly6C^+$) under SC and WD feeding. (n=5). **(C)** Differentiated macrophages in blood and liver (Kupffer cells: $CD45^+Hoechst^-Ly6G^-CD11b^+F4/80^{++}$) under SC and conditions of experimental steatohepatitis (WD). (n=5), (*** p<0.001) **(D)** Blood and intrahepatic neutrophils ($CD45^+Ly6G^+$) under SC and WD treatment. (n=5), (** p<0.01). **(E)** Blood and hepatic NK cell ($CD45^+Hoechst^-CD3^-NK1.1^+$) numbers after SC and WD feeding for 24 weeks. (n=5). **(F)** Blood and hepatic NKT cell ($CD45^+CD3^+NK1.1^+CD8^+$) numbers under SC and WD. (n=5), (** p<0.01).

Supplementary Figure 5: Quantification of a multiplex staining for T cell activation markers. (A) Quantification of PD1 positive cells in livers of SC and WD fed animals for 24 weeks. **(B)** Cell positivity of $PD1^+$ $CD8^+$ and $PD1^+$ $CD4^+$ double positive cells after 24 weeks treatment with SC or WD. **(C)** Quantification of CD107a positive cells in livers of SC and WD fed animals for 24 weeks. **(D)** Cell positivity of $CD107a^+$ $CD8^+$ and $CD107a^+$ $CD4^+$ double positive cells after 24 weeks treatment with SC or WD.

Supplementary Figure 6: Pattern recognition learning algorithm supporting a tissue and cell classifier. Images of multiplexed immunofluorescence stained liver sections (DAPI, PD1, CD107a, CD4, CD8) of **(A)** SC and **(B)** WD fed mice. Magnification 200x. Scans of the multispectral image acquisition and analysis showing the spectrally unmixed image, cell segmentation, phenotyping and scoring of a SC and a WD fed mouse.

Supplementary Figure 7: PD1 expression on $CD11b^+$ cells. Representative images of CD11b/PD1 stained liver sections of **(A)** SC and **(B)** WD fed animals for 24 weeks. Scale bars 100 µm, magnification 200x. Nuclei are stained with Dapi (blue), CD11b (green) PD1 (red). $CD11b^+/PD1^+$ double positive cells are marked with white arrows.

Supplementary Table 1

Product No.	D09100301			SC diet AIN 93M
	gm%	kcal%		kcal%
Protein	22	20	Protein	13
Carbohydrate	45	40	Carbohydrates	77
Fat	20	40	Fat	10
Total		100	Total	100
kcal/gm	4,5		kcal/kg	3,735
**Igredient**	**gm**	**kcal**		
Casein, 80 Mesh	200	800		
L-Cystine	3	12		
Maltodextrin 10	100	400		
Fructose	200	800		
Sucrose	96	384		
Cellulose, BW 200	50	0		
Soybean Oil	25	225		
**Primex Shortening**	**135**	1215		
**Primex, 101650, non trans fat**	**0**	0		
**Lard**	20	180		
Mineral Mix S10026	10	0	**Minerals**	**%**
DiCalcium Phosphate	13	0	Calcium	0,55
Calcium Carbonate	5,5	0	Phosphorus	0,36
Potassium Citrate, 1 H2O	16,5	0	Ca/P	1,55:1
			Sodium	0,16
Vitamin Mix V10001	10	40	Magnesium	0,09
Choline Bitartrate	2	0	Potassium	0,54
**Cholesterol**	**18**	0		
FD&C Yellow Dye #5	0,05	0		
FD&C Red Dye #40	0	0		
FD&C Blue Dye #1	0	0		
**Total**	**904,05**	**4056**		
	**WD**			**SC diet**
	Taken from Broogarden			Taken from Ssniff

Supplementary Table 2

Baseline patient demographic and disease characteristics for flow cytometric analysis

n=10	
Age [years], median [range]	62 [43-74]
Sex, n [%]	
Males	8 [80]
Females	2 [20]
Etiology, n [%]	
Control	5 [50]
NASH	5 [50]
AST [U/L], median [range]	34 [12-103]
ALT [U/L], median [range]	18 [5-31]
γGT [U/L], median [range]	109 [18-261]
INR, median [range]	1.23 [0.89-2.35]
Alcaline Phosphatese [U/L], median [range]	98 [54-262]
Total Billirubin [mg/dL], median [range]	2.24 [0.20-10.17]

Supplementary Figure 1

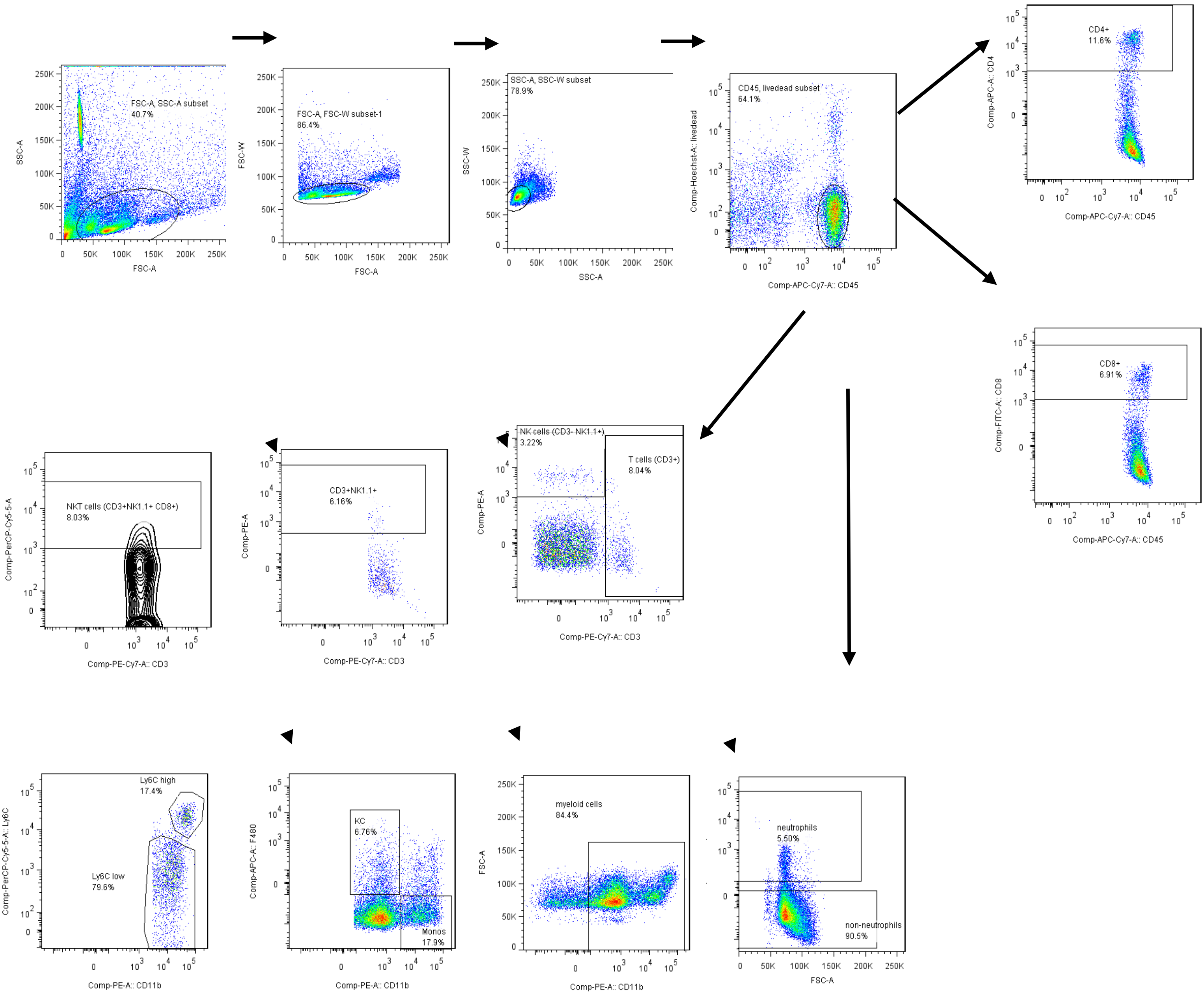

Supplementary Figure 2

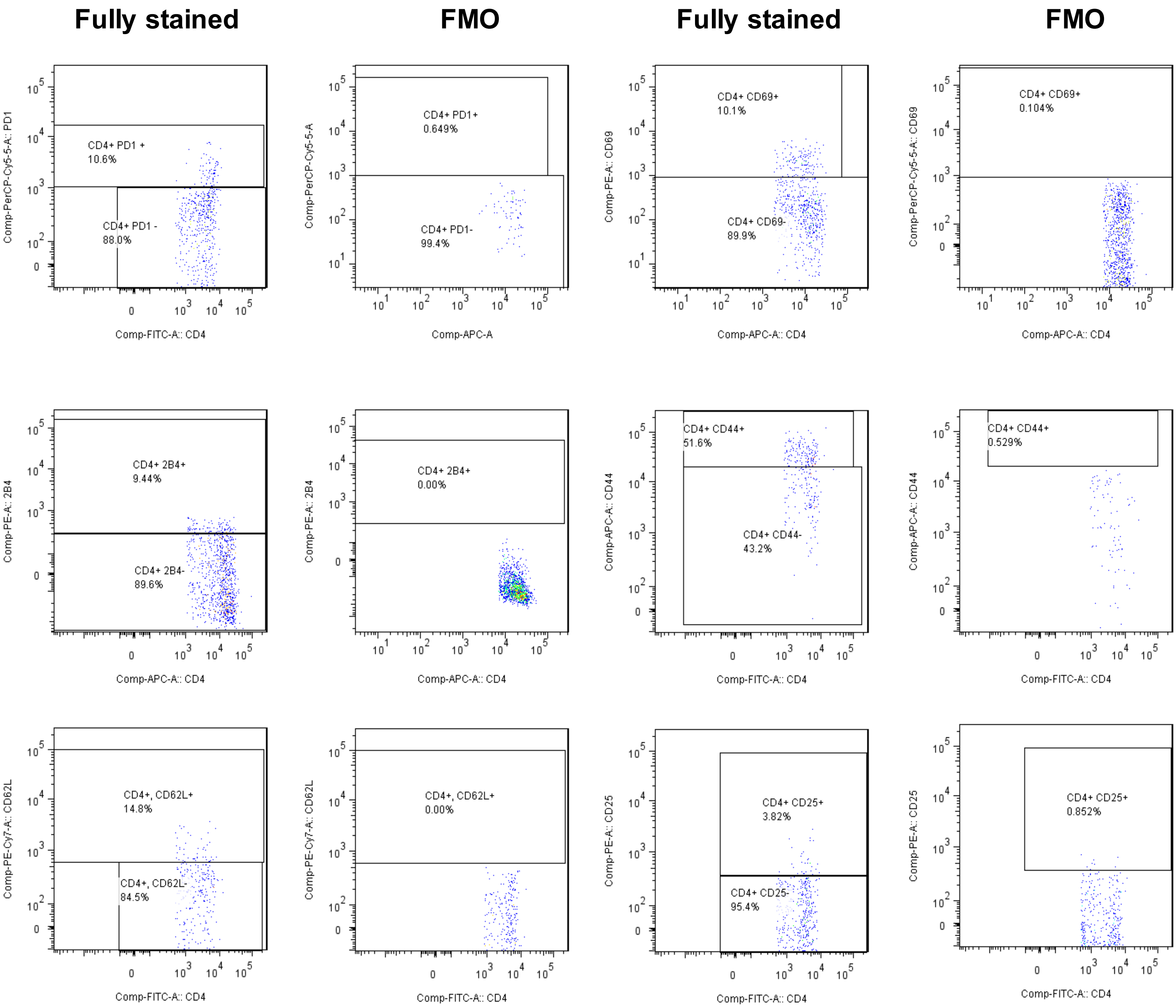

Supplementary Figure 3

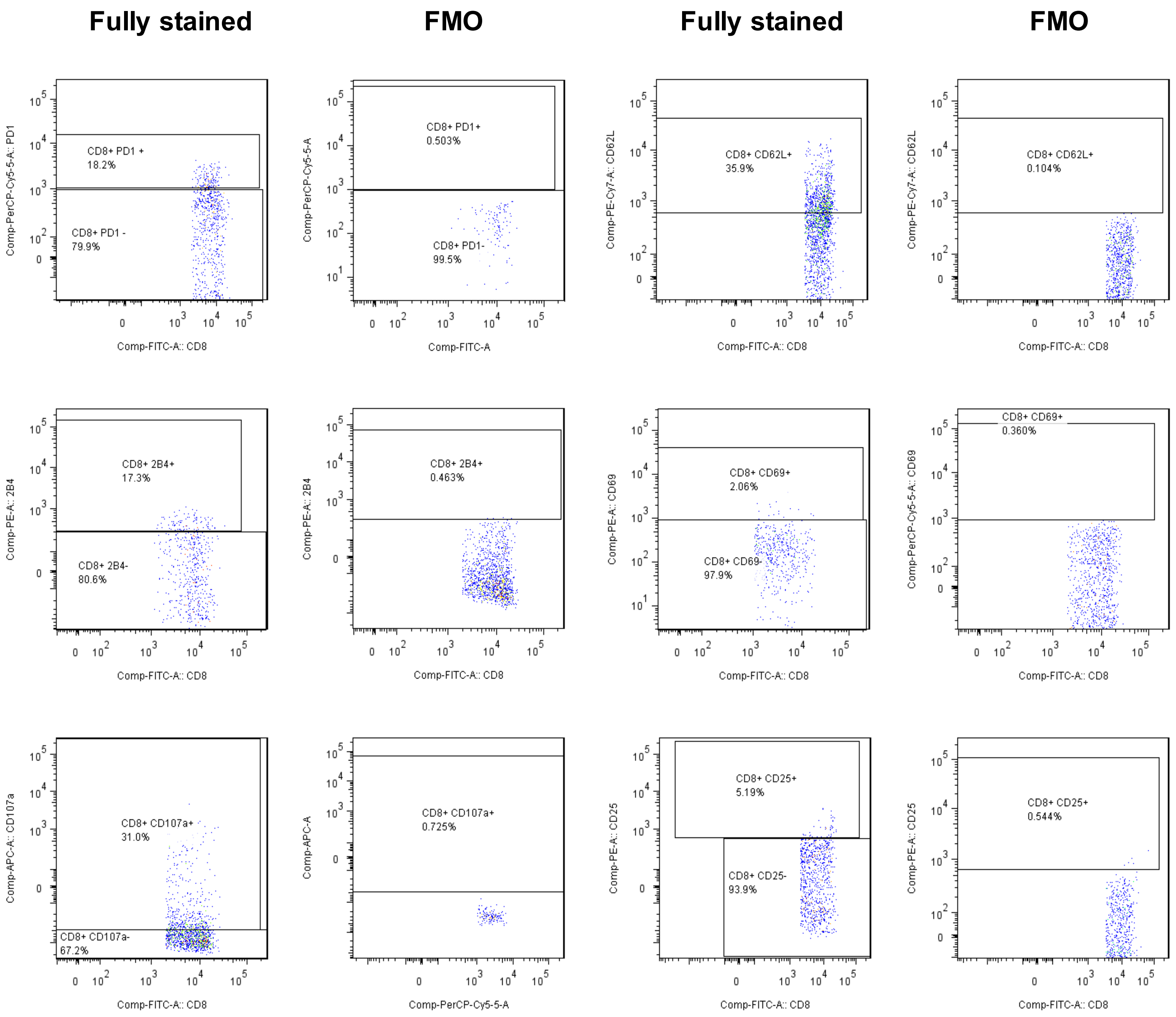

Supplementary Figure 4

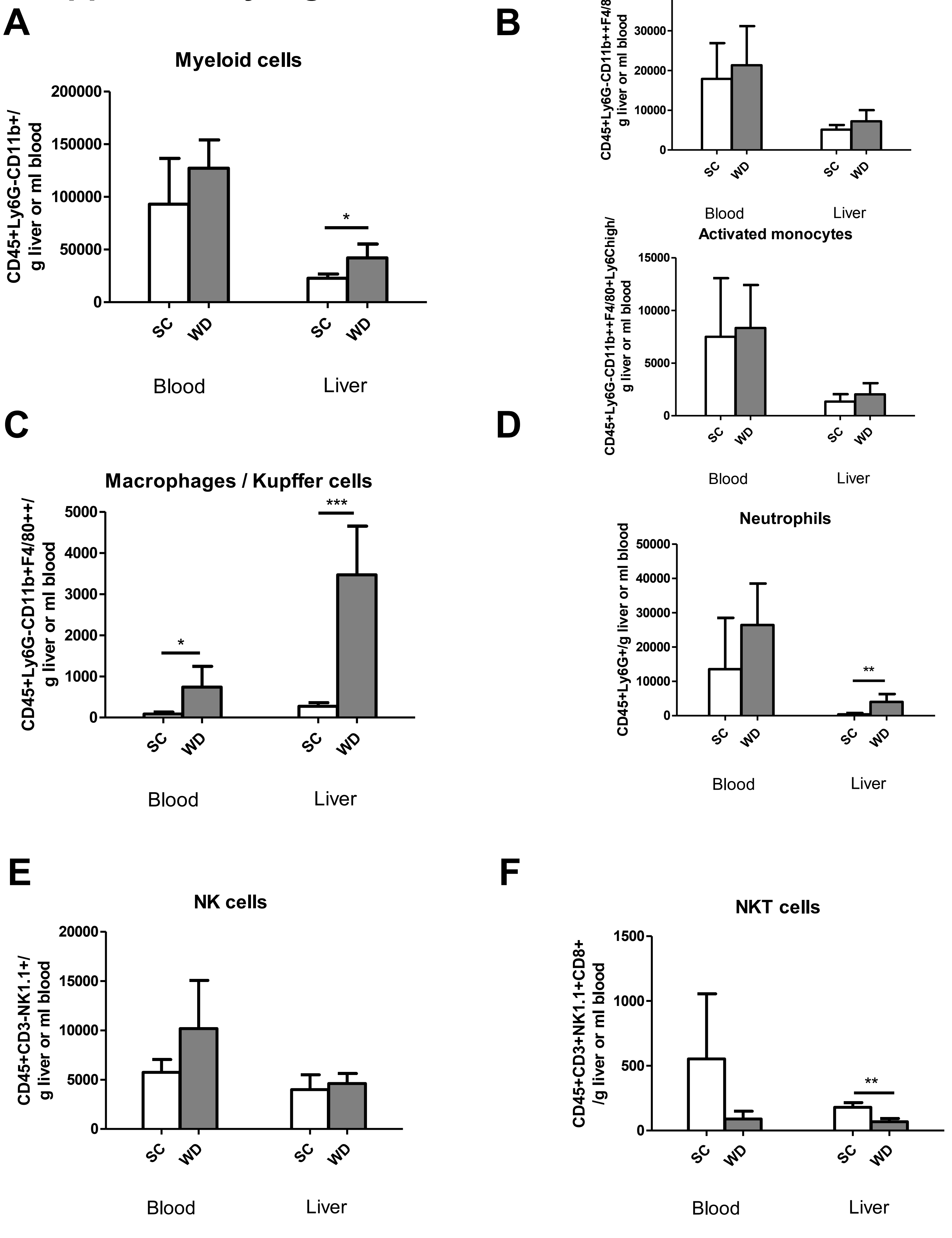

Supplementary Figure 5

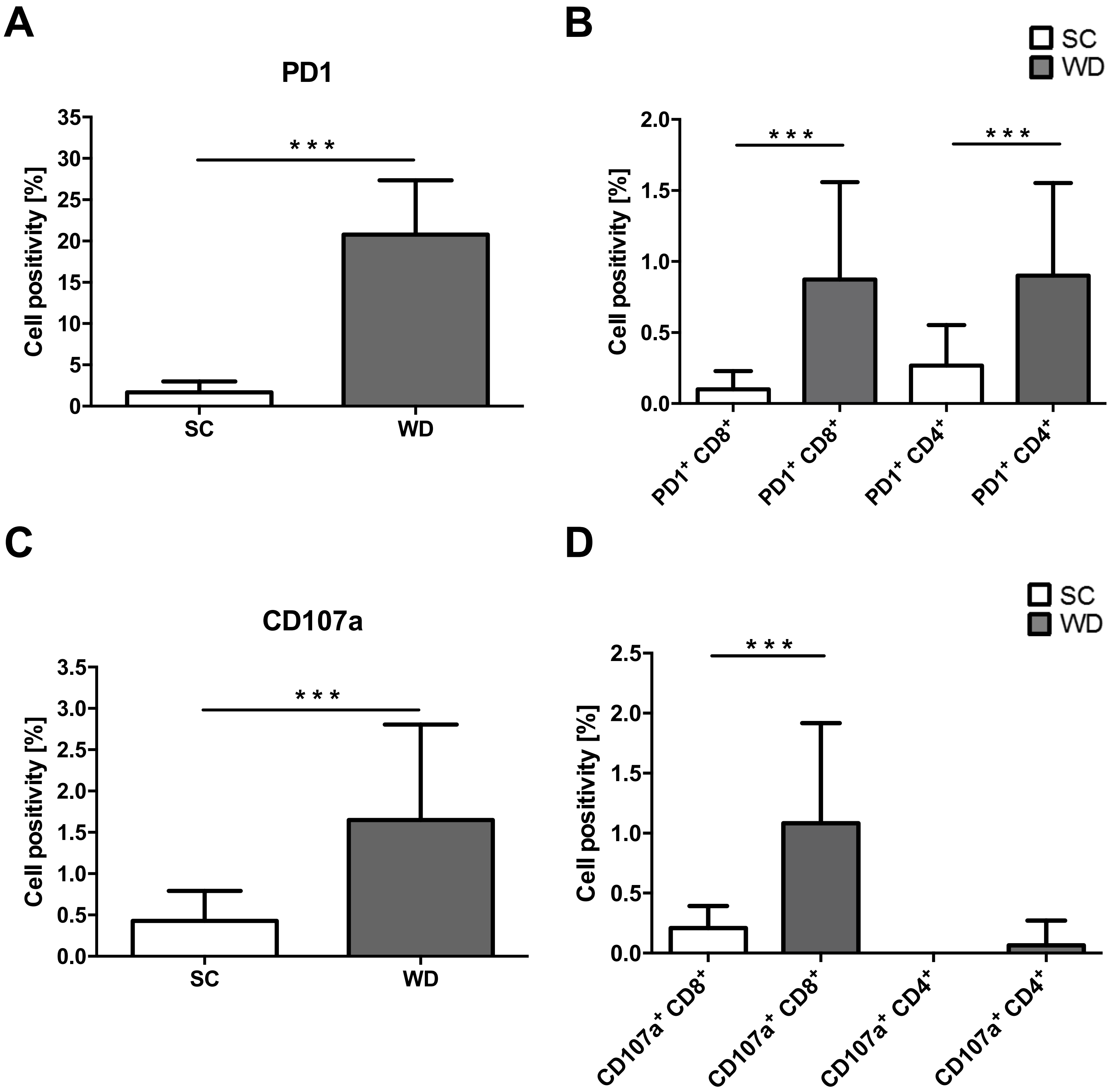

Supplementary Figure 6

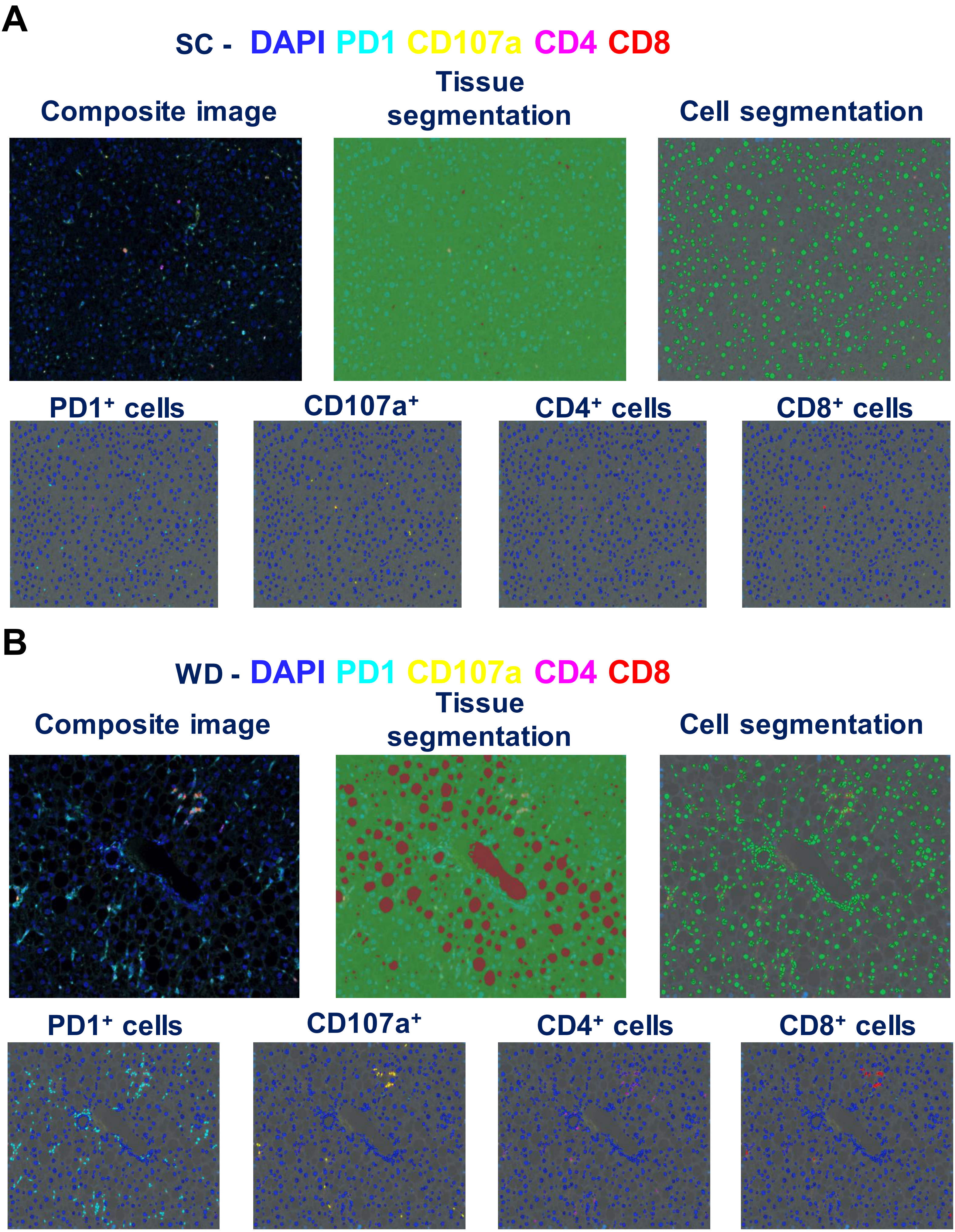

Supplementary Figure 7

SC WD

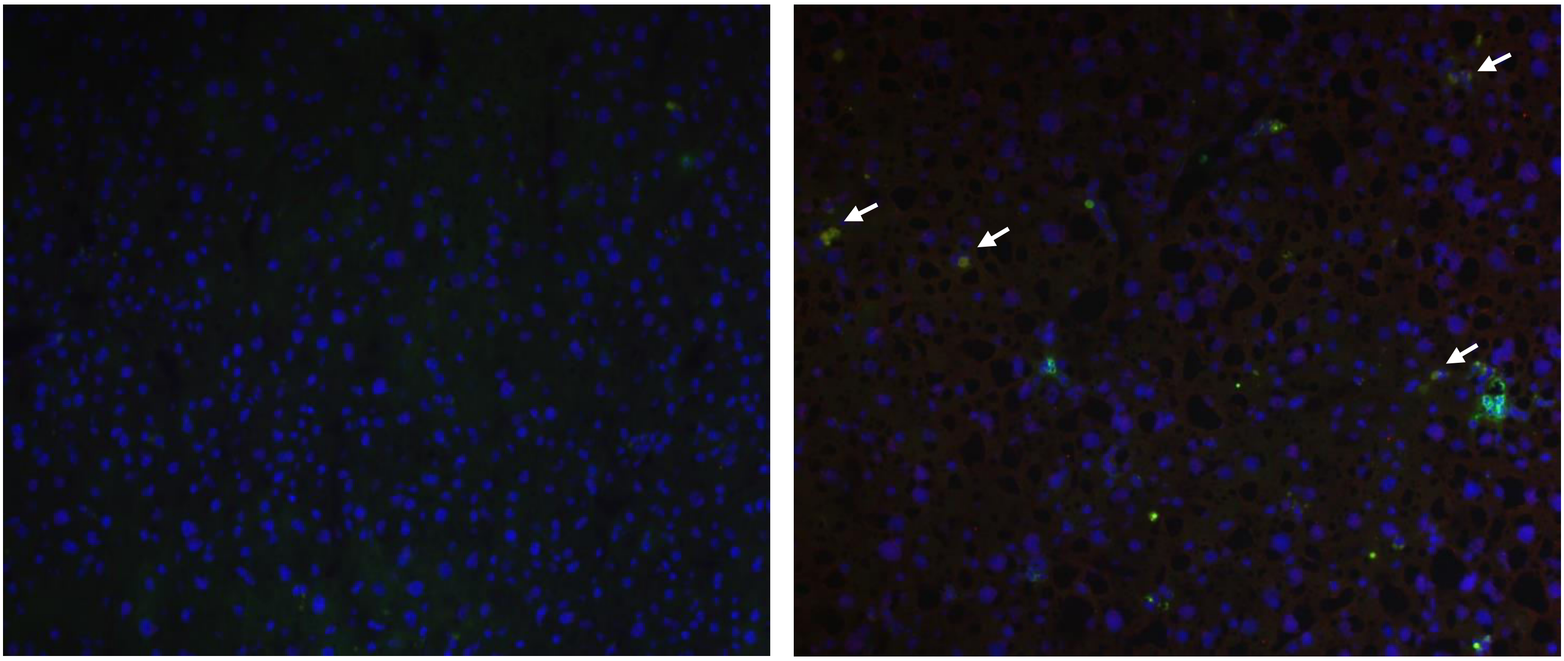

Supplement: Supplementary file 1 [file Data_Sheet_1.pdf]
